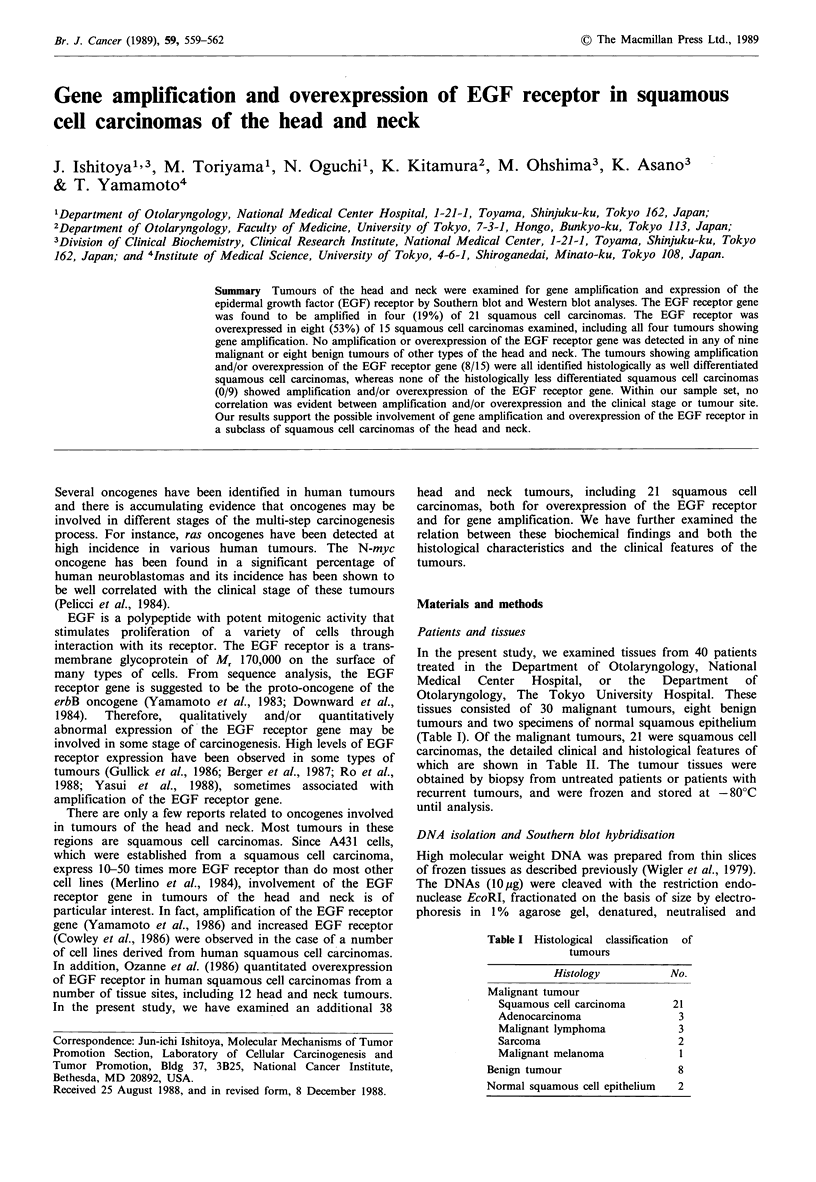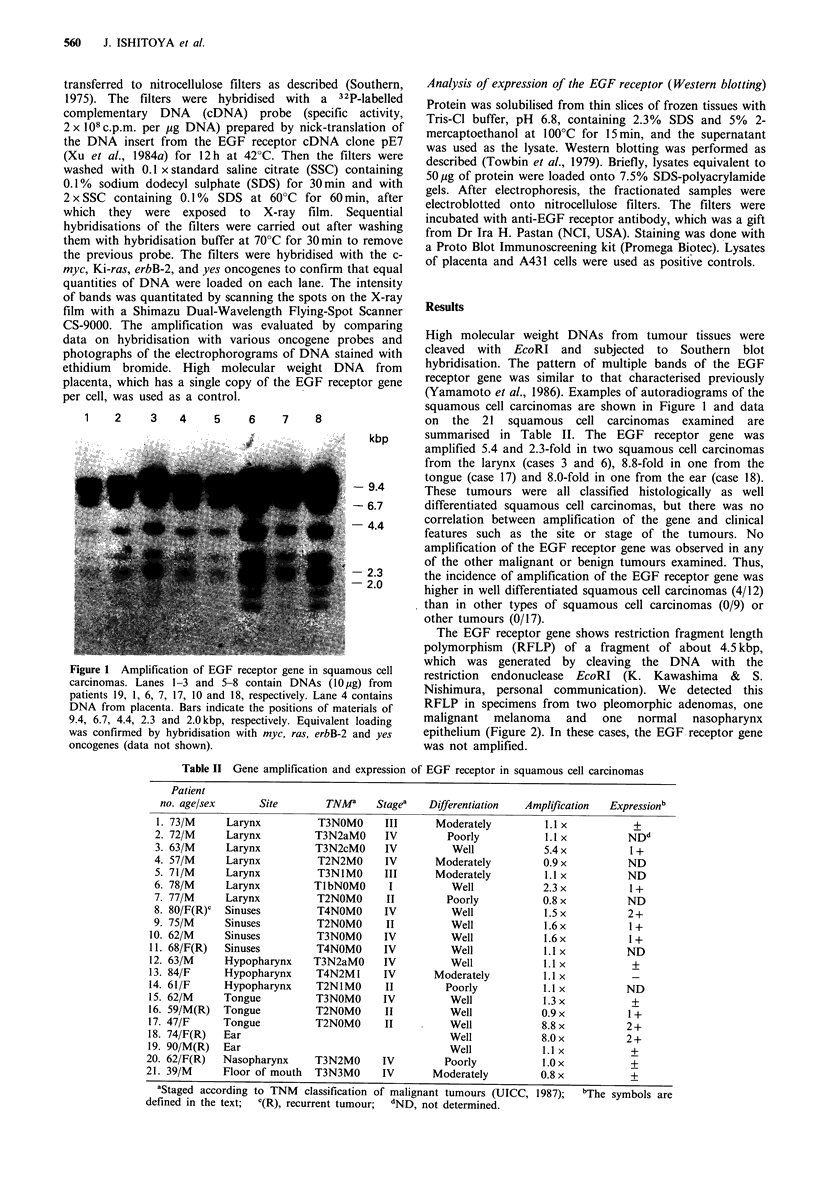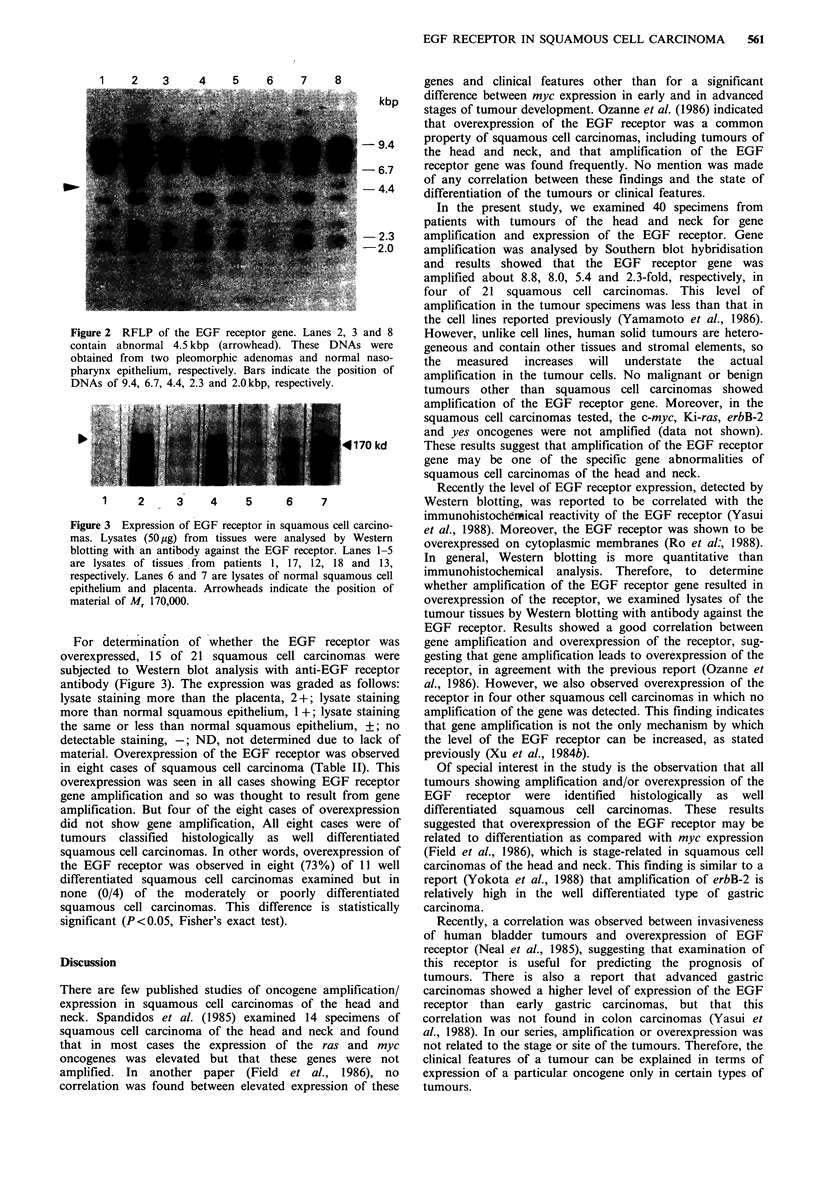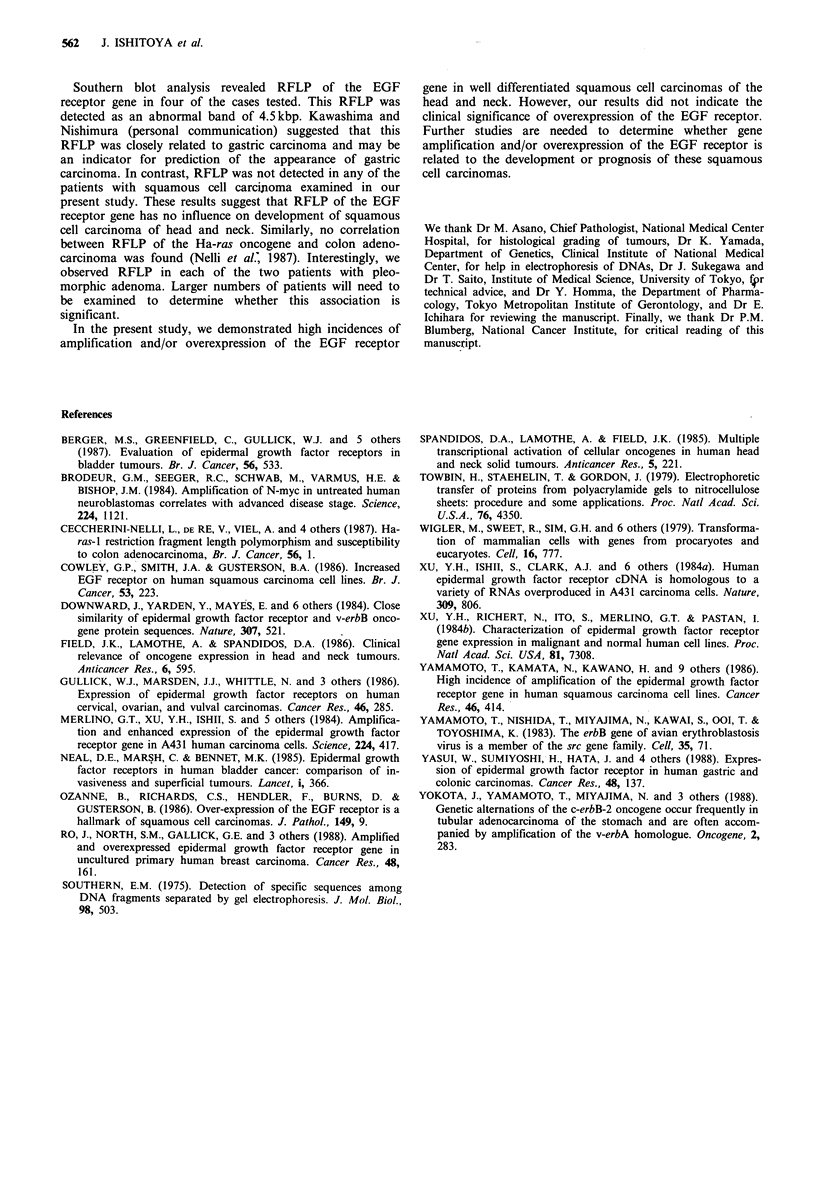# Gene amplification and overexpression of EGF receptor in squamous cell carcinomas of the head and neck.

**DOI:** 10.1038/bjc.1989.113

**Published:** 1989-04

**Authors:** J. Ishitoya, M. Toriyama, N. Oguchi, K. Kitamura, M. Ohshima, K. Asano, T. Yamamoto

**Affiliations:** Department of Otolaryngology, National Medical Center Hospital, Tokyo.

## Abstract

**Images:**


Br. J. Cancer (1989), 59, 559-562 © The Macmillan Press Ltd., 1989

### Gene amplification and overexpression of EGF receptor in squamous cell carcinomas of the head and neck

J. Ishitoya[1,3], M. Toriyama[1], N. Oguchi[1], K. Kitamura[2], M. Ohshima[3], K. Asano[3] & T. Yamamoto[4]

[1]*Department of Otolaryngology, National Medical Center Hospital, 1-21-1, Toyama, Shinjuku-ku, Tokyo 162, Japan;* [2]*Department of Otolaryngology, Faculty of Medicine, University of Tokyo, 7-3-1, Hongo, Bunkyo-ku, Tokyo 113, Japan;* [3]*Division of Clinical Biochemistry, Clinical Research Institute, National Medical Center, 1-21-1, Toyama, Shinjuku-ku, Tokyo 162, Japan; and* [4]*Institute of Medical Science, University of Tokyo, 4-6-1, Shiroganedai, Minato-ku, Tokyo 108, Japan.*

**Summary** Tumours of the head and neck were examined for gene amplification and expression of the epidermal growth factor (EGF) receptor by Southern blot and Western blot analyses. The EGF receptor gene was found to be amplified in four (19%) of 21 squamous cell carcinomas. The EGF receptor was overexpressed in eight (53%) of 15 squamous cell carcinomas examined, including all four tumours showing gene amplification. No amplification or overexpression of the EGF receptor gene was detected in any of nine malignant or eight benign tumours of other types of the head and neck. The tumours showing amplification and/or overexpression of the EGF receptor gene (8/15) were all identified histologically as well differentiated squamous cell carcinomas, whereas none of the histologically less differentiated squamous cell carcinomas (0/9) showed amplification and/or overexpression of the EGF receptor gene. Within our sample set, no correlation was evident between amplification and/or overexpression and the clinical stage or tumour site. Our results support the possible involvement of gene amplification and overexpression of the EGF receptor in a subclass of squamous cell carcinomas of the head and neck.

Several oncogenes have been identified in human tumours and there is accumulating evidence that oncogenes may be involved in different stages of the multi-step carcinogenesis process. For instance, *ras* oncogenes have been detected at high incidence in various human tumours. The N-*myc* oncogene has been found in a significant percentage of human neuroblastomas and its incidence has been shown to be well correlated with the clinical stage of these tumours (Pelicci *et al.*, 1984).

EGF is a polypeptide with potent mitogenic activity that stimulates proliferation of a variety of cells through interaction with its receptor. The EGF receptor is a transmembrane glycoprotein of $M_r$ 170,000 on the surface of many types of cells. From sequence analysis, the EGF receptor gene is suggested to be the proto-oncogene of the *erb*B oncogene (Yamamoto *et al.*, 1983; Downward *et al.*, 1984). Therefore, qualitatively and/or quantitatively abnormal expression of the EGF receptor gene may be involved in some stage of carcinogenesis. High levels of EGF receptor expression have been observed in some types of tumours (Gullick *et al.*, 1986; Berger *et al.*, 1987; Ro *et al.*, 1988; Yasui *et al.*, 1988), sometimes associated with amplification of the EGF receptor gene.

There are only a few reports related to oncogenes involved in tumours of the head and neck. Most tumours in these regions are squamous cell carcinomas. Since A431 cells, which were established from a squamous cell carcinoma, express 10–50 times more EGF receptor than do most other cell lines (Merlino *et al.*, 1984), involvement of the EGF receptor gene in tumours of the head and neck is of particular interest. In fact, amplification of the EGF receptor gene (Yamamoto *et al.*, 1986) and increased EGF receptor (Cowley *et al.*, 1986) were observed in the case of a number of cell lines derived from human squamous cell carcinomas. In addition, Ozanne *et al.* (1986) quantitated overexpression of EGF receptor in human squamous cell carcinomas from a number of tissue sites, including 12 head and neck tumours. In the present study, we have examined an additional 38 head and neck tumours, including 21 squamous cell carcinomas, both for overexpression of the EGF receptor and for gene amplification. We have further examined the relation between these biochemical findings and both the histological characteristics and the clinical features of the tumours.

Correspondence: Jun-ichi Ishitoya, Molecular Mechanisms of Tumor Promotion Section, Laboratory of Cellular Carcinogenesis and Tumor Promotion, Bldg 37, 3B25, National Cancer Institute, Bethesda, MD 20892, USA.
Received 25 August 1988, and in revised form, 8 December 1988.

### Materials and methods

### *Patients and tissues*

In the present study, we examined tissues from 40 patients treated in the Department of Otolaryngology, National Medical Center Hospital, or the Department of Otolaryngology, The Tokyo University Hospital. These tissues consisted of 30 malignant tumours, eight benign tumours and two specimens of normal squamous epithelium (Table I). Of the malignant tumours, 21 were squamous cell carcinomas, the detailed clinical and histological features of which are shown in Table II. The tumour tissues were obtained by biopsy from untreated patients or patients with recurrent tumours, and were frozen and stored at −80°C until analysis.

### *DNA isolation and Southern blot hybridisation*

High molecular weight DNA was prepared from thin slices of frozen tissues as described previously (Wigler *et al.*, 1979). The DNAs (10 μg) were cleaved with the restriction endonuclease *Eco*RI, fractionated on the basis of size by electrophoresis in 1% agarose gel, denatured, neutralised and

**Table I** Histological classification of tumours

| *Histology* | *No.* |
|---|---|
| Malignant tumour | |
| Squamous cell carcinoma | 21 |
| Adenocarcinoma | 3 |
| Malignant lymphoma | 3 |
| Sarcoma | 2 |
| Malignant melanoma | 1 |
| Benign tumour | 8 |
| Normal squamous cell epithelium | 2 |

transferred to nitrocellulose filters as described (Southern, 1975). The filters were hybridised with a $^{32}P$-labelled complementary DNA (cDNA) probe (specific activity, $2 \times 10^8$ c.p.m. per $\mu$g DNA) prepared by nick-translation of the DNA insert from the EGF receptor cDNA clone pE7 (Xu *et al.*, 1984*a*) for 12 h at 42°C. Then the filters were washed with 0.1 × standard saline citrate (SSC) containing 0.1% sodium dodecyl sulphate (SDS) for 30 min and with 2 × SSC containing 0.1% SDS at 60°C for 60 min, after which they were exposed to X-ray film. Sequential hybridisations of the filters were carried out after washing them with hybridisation buffer at 70°C for 30 min to remove the previous probe. The filters were hybridised with the c-*myc*, Ki-*ras*, *erb*B-2, and *yes* oncogenes to confirm that equal quantities of DNA were loaded on each lane. The intensity of bands was quantitated by scanning the spots on the X-ray film with a Shimazu Dual-Wavelength Flying-Spot Scanner CS-9000. The amplification was evaluated by comparing data on hybridisation with various oncogene probes and photographs of the electrophorograms of DNA stained with ethidium bromide. High molecular weight DNA from placenta, which has a single copy of the EGF receptor gene per cell, was used as a control.

**Figure 1** Amplification of EGF receptor gene in squamous cell carcinomas. Lanes 1–3 and 5–8 contain DNAs (10 $\mu$g) from patients 19, 1, 6, 7, 17, 10 and 18, respectively. Lane 4 contains DNA from placenta. Bars indicate the positions of materials of 9.4, 6.7, 4.4, 2.3 and 2.0 kbp, respectively. Equivalent loading was confirmed by hybridisation with *myc*, *ras*, *erb*B-2 and *yes* oncogenes (data not shown).

*Analysis of expression of the EGF receptor* (*Western blotting*)

Protein was solubilised from thin slices of frozen tissues with Tris-Cl buffer, pH 6.8, containing 2.3% SDS and 5% 2-mercaptoethanol at 100°C for 15 min, and the supernatant was used as the lysate. Western blotting was performed as described (Towbin *et al.*, 1979). Briefly, lysates equivalent to 50 $\mu$g of protein were loaded onto 7.5% SDS-polyacrylamide gels. After electrophoresis, the fractionated samples were electroblotted onto nitrocellulose filters. The filters were incubated with anti-EGF receptor antibody, which was a gift from Dr Ira H. Pastan (NCI, USA). Staining was done with a Proto Blot Immunoscreening kit (Promega Biotec). Lysates of placenta and A431 cells were used as positive controls.

### Results

High molecular weight DNAs from tumour tissues were cleaved with *Eco*RI and subjected to Southern blot hybridisation. The pattern of multiple bands of the EGF receptor gene was similar to that characterised previously (Yamamoto *et al.*, 1986). Examples of autoradiograms of the squamous cell carcinomas are shown in Figure 1 and data on the 21 squamous cell carcinomas examined are summarised in Table II. The EGF receptor gene was amplified 5.4 and 2.3-fold in two squamous cell carcinomas from the larynx (cases 3 and 6), 8.8-fold in one from the tongue (case 17) and 8.0-fold in one from the ear (case 18). These tumours were all classified histologically as well differentiated squamous cell carcinomas, but there was no correlation between amplification of the gene and clinical features such as the site or stage of the tumours. No amplification of the EGF receptor gene was observed in any of the other malignant or benign tumours examined. Thus, the incidence of amplification of the EGF receptor gene was higher in well differentiated squamous cell carcinomas (4/12) than in other types of squamous cell carcinomas (0/9) or other tumours (0/17).

The EGF receptor gene shows restriction fragment length polymorphism (RFLP) of a fragment of about 4.5 kbp, which was generated by cleaving the DNA with the restriction endonuclease *Eco*RI (K. Kawashima & S. Nishimura, personal communication). We detected this RFLP in specimens from two pleomorphic adenomas, one malignant melanoma and one normal nasopharynx epithelium (Figure 2). In these cases, the EGF receptor gene was not amplified.

**Table II** Gene amplification and expression of EGF receptor in squamous cell carcinomas

| *Patient no. age/sex* | *Site* | *TNM*[a] | *Stage*[a] | *Differentiation* | *Amplification* | *Expression*[b] |
|---|---|---|---|---|---|---|
| 1. 73/M | Larynx | T3N0M0 | III | Moderately | 1.1 × | ± |
| 2. 72/M | Larynx | T3N2aM0 | IV | Poorly | 1.1 × | ND[d] |
| 3. 63/M | Larynx | T3N2cM0 | IV | Well | 5.4 × | 1+ |
| 4. 57/M | Larynx | T2N2M0 | IV | Moderately | 0.9 × | ND |
| 5. 71/M | Larynx | T3N1M0 | III | Moderately | 1.1 × | ND |
| 6. 78/M | Larynx | T1bN0M0 | I | Well | 2.3 × | 1+ |
| 7. 77/M | Larynx | T2N0M0 | II | Poorly | 0.8 × | ND |
| 8. 80/F(R)[c] | Sinuses | T4N0M0 | IV | Well | 1.5 × | 2+ |
| 9. 75/M | Sinuses | T2N0M0 | II | Well | 1.6 × | 1+ |
| 10. 62/M | Sinuses | T3N0M0 | IV | Well | 1.6 × | 1+ |
| 11. 68/F(R) | Sinuses | T4N0M0 | IV | Well | 1.1 × | ND |
| 12. 63/M | Hypopharynx | T3N2aM0 | IV | Well | 1.1 × | ± |
| 13. 84/F | Hypopharynx | T4N2M1 | IV | Moderately | 1.1 × | – |
| 14. 61/F | Hypopharynx | T2N1M0 | II | Poorly | 1.1 × | ND |
| 15. 62/M | Tongue | T3N0M0 | IV | Well | 1.3 × | ± |
| 16. 59/M(R) | Tongue | T2N0M0 | II | Well | 0.9 × | 1+ |
| 17. 47/F | Tongue | T2N0M0 | II | Well | 8.8 × | 2+ |
| 18. 74/F(R) | Ear | | | Well | 8.0 × | 2+ |
| 19. 90/M(R) | Ear | | | Well | 1.1 × | ± |
| 20. 62/F(R) | Nasopharynx | T3N2M0 | IV | Poorly | 1.0 × | ± |
| 21. 39/M | Floor of mouth | T3N3M0 | IV | Moderately | 0.8 × | ± |

[a]Staged according to TNM classification of malignant tumours (UICC, 1987); [b]The symbols are defined in the text; [c](R), recurrent tumour; [d]ND, not determined.

**Figure 2** RFLP of the EGF receptor gene. Lanes 2, 3 and 8 contain abnormal 4.5 kbp (arrowhead). These DNAs were obtained from two pleomorphic adenomas and normal nasopharynx epithelium, respectively. Bars indicate the position of DNAs of 9.4, 6.7, 4.4, 2.3 and 2.0 kbp, respectively.

**Figure 3** Expression of EGF receptor in squamous cell carcinomas. Lysates (50 $\mu$g) from tissues were analysed by Western blotting with an antibody against the EGF receptor. Lanes 1–5 are lysates of tissues from patients 1, 17, 12, 18 and 13, respectively. Lanes 6 and 7 are lysates of normal squamous cell epithelium and placenta. Arrowheads indicate the position of material of $M_r$ 170,000.

For determination of whether the EGF receptor was overexpressed, 15 of 21 squamous cell carcinomas were subjected to Western blot analysis with anti-EGF receptor antibody (Figure 3). The expression was graded as follows: lysate staining more than the placenta, 2+; lysate staining more than normal squamous epithelium, 1+; lysate staining the same or less than normal squamous epithelium, $\pm$; no detectable staining, $-$; ND, not determined due to lack of material. Overexpression of the EGF receptor was observed in eight cases of squamous cell carcinoma (Table II). This overexpression was seen in all cases showing EGF receptor gene amplification and so was thought to result from gene amplification. But four of the eight cases of overexpression did not show gene amplification, All eight cases were of tumours classified histologically as well differentiated squamous cell carcinomas. In other words, overexpression of the EGF receptor was observed in eight (73%) of 11 well differentiated squamous cell carcinomas examined but in none (0/4) of the moderately or poorly differentiated squamous cell carcinomas. This difference is statistically significant ($P<0.05$, Fisher's exact test).

### Discussion

There are few published studies of oncogene amplification/expression in squamous cell carcinomas of the head and neck. Spandidos *et al.* (1985) examined 14 specimens of squamous cell carcinoma of the head and neck and found that in most cases the expression of the *ras* and *myc* oncogenes was elevated but that these genes were not amplified. In another paper (Field *et al.*, 1986), no correlation was found between elevated expression of these genes and clinical features other than for a significant difference between *myc* expression in early and in advanced stages of tumour development. Ozanne *et al.* (1986) indicated that overexpression of the EGF receptor was a common property of squamous cell carcinomas, including tumours of the head and neck, and that amplification of the EGF receptor gene was found frequently. No mention was made of any correlation between these findings and the state of differentiation of the tumours or clinical features.

In the present study, we examined 40 specimens from patients with tumours of the head and neck for gene amplification and expression of the EGF receptor. Gene amplification was analysed by Southern blot hybridisation and results showed that the EGF receptor gene was amplified about 8.8, 8.0, 5.4 and 2.3-fold, respectively, in four of 21 squamous cell carcinomas. This level of amplification in the tumour specimens was less than that in the cell lines reported previously (Yamamoto *et al.*, 1986). However, unlike cell lines, human solid tumours are heterogeneous and contain other tissues and stromal elements, so the measured increases will understate the actual amplification in the tumour cells. No malignant or benign tumours other than squamous cell carcinomas showed amplification of the EGF receptor gene. Moreover, in the squamous cell carcinomas tested, the c-*myc*, Ki-*ras*, *erb*B-2 and *yes* oncogenes were not amplified (data not shown). These results suggest that amplification of the EGF receptor gene may be one of the specific gene abnormalities of squamous cell carcinomas of the head and neck.

Recently the level of EGF receptor expression, detected by Western blotting, was reported to be correlated with the immunohistochemical reactivity of the EGF receptor (Yasui *et al.*, 1988). Moreover, the EGF receptor was shown to be overexpressed on cytoplasmic membranes (Ro *et al.*, 1988). In general, Western blotting is more quantitative than immunohistochemical analysis. Therefore, to determine whether amplification of the EGF receptor gene resulted in overexpression of the receptor, we examined lysates of the tumour tissues by Western blotting with antibody against the EGF receptor. Results showed a good correlation between gene amplification and overexpression of the receptor, suggesting that gene amplification leads to overexpression of the receptor, in agreement with the previous report (Ozanne *et al.*, 1986). However, we also observed overexpression of the receptor in four other squamous cell carcinomas in which no amplification of the gene was detected. This finding indicates that gene amplification is not the only mechanism by which the level of the EGF receptor can be increased, as stated previously (Xu *et al.*, 1984*b*).

Of special interest in the study is the observation that all tumours showing amplification and/or overexpression of the EGF receptor were identified histologically as well differentiated squamous cell carcinomas. These results suggested that overexpression of the EGF receptor may be related to differentiation as compared with *myc* expression (Field *et al.*, 1986), which is stage-related in squamous cell carcinomas of the head and neck. This finding is similar to a report (Yokota *et al.*, 1988) that amplification of *erb*B-2 is relatively high in the well differentiated type of gastric carcinoma.

Recently, a correlation was observed between invasiveness of human bladder tumours and overexpression of EGF receptor (Neal *et al.*, 1985), suggesting that examination of this receptor is useful for predicting the prognosis of tumours. There is also a report that advanced gastric carcinomas showed a higher level of expression of the EGF receptor than early gastric carcinomas, but that this correlation was not found in colon carcinomas (Yasui *et al.*, 1988). In our series, amplification or overexpression was not related to the stage or site of the tumours. Therefore, the clinical features of a tumour can be explained in terms of expression of a particular oncogene only in certain types of tumours.

Southern blot analysis revealed RFLP of the EGF receptor gene in four of the cases tested. This RFLP was detected as an abnormal band of 4.5 kbp. Kawashima and Nishimura (personal communication) suggested that this RFLP was closely related to gastric carcinoma and may be an indicator for prediction of the appearance of gastric carcinoma. In contrast, RFLP was not detected in any of the patients with squamous cell carcinoma examined in our present study. These results suggest that RFLP of the EGF receptor gene has no influence on development of squamous cell carcinoma of head and neck. Similarly, no correlation between RFLP of the Ha-*ras* oncogene and colon adenocarcinoma was found (Nelli *et al.*, 1987). Interestingly, we observed RFLP in each of the two patients with pleomorphic adenoma. Larger numbers of patients will need to be examined to determine whether this association is significant.

In the present study, we demonstrated high incidences of amplification and/or overexpression of the EGF receptor gene in well differentiated squamous cell carcinomas of the head and neck. However, our results did not indicate the clinical significance of overexpression of the EGF receptor. Further studies are needed to determine whether gene amplification and/or overexpression of the EGF receptor is related to the development or prognosis of these squamous cell carcinomas.

We thank Dr M. Asano, Chief Pathologist, National Medical Center Hospital, for histological grading of tumours, Dr K. Yamada, Department of Genetics, Clinical Institute of National Medical Center, for help in electrophoresis of DNAs, Dr J. Sukegawa and Dr T. Saito, Institute of Medical Science, University of Tokyo, for technical advice, and Dr Y. Homma, the Department of Pharmacology, Tokyo Metropolitan Institute of Gerontology, and Dr E. Ichihara for reviewing the manuscript. Finally, we thank Dr P.M. Blumberg, National Cancer Institute, for critical reading of this manuscript.

### References

BERGER, M.S., GREENFIELD, C., GULLICK, W.J. and 5 others (1987). Evaluation of epidermal growth factor receptors in bladder tumours. *Br. J. Cancer*, **56**, 533.

BRODEUR, G.M., SEEGER, R.C., SCHWAB, M., VARMUS, H.E. & BISHOP, J.M. (1984). Amplification of N-myc in untreated human neuroblastomas correlates with advanced disease stage. *Science*, **224**, 1121.

CECCHERINI-NELLI, L., DE RE, V., VIEL, A. and 4 others (1987). Ha-*ras*-1 restriction fragment length polymorphism and susceptibility to colon adenocarcinoma, *Br. J. Cancer*, **56**, 1.

COWLEY, G.P., SMITH, J.A. & GUSTERSON, B.A. (1986). Increased EGF receptor on human squamous carcinoma cell lines. *Br. J. Cancer*, **53**, 223.

DOWNWARD, J., YARDEN, Y., MAYES, E. and 6 others (1984). Close similarity of epidermal growth factor receptor and v-*erb*B oncogene protein sequences. *Nature*, **307**, 521.

FIELD, J.K., LAMOTHE, A. & SPANDIDOS, D.A. (1986). Clinical relevance of oncogene expression in head and neck tumours. *Anticancer Res.*, **6**, 595.

GULLICK, W.J., MARSDEN, J.J., WHITTLE, N. and 3 others (1986). Expression of epidermal growth factor receptors on human cervical, ovarian, and vulval carcinomas. *Cancer Res.*, **46**, 285.

MERLINO, G.T., XU, Y.H., ISHII, S. and 5 others (1984). Amplification and enhanced expression of the epidermal growth factor receptor gene in A431 human carcinoma cells. *Science*, **224**, 417.

NEAL, D.E., MARSH, C. & BENNET, M.K. (1985). Epidermal growth factor receptors in human bladder cancer: comparison of invasiveness and superficial tumours. *Lancet*, **i**, 366.

OZANNE, B., RICHARDS, C.S., HENDLER, F., BURNS, D. & GUSTERSON, B. (1986). Over-expression of the EGF receptor is a hallmark of squamous cell carcinomas. *J. Pathol.*, **149**, 9.

RO, J., NORTH, S.M., GALLICK, G.E. and 3 others (1988). Amplified and overexpressed epidermal growth factor receptor gene in uncultured primary human breast carcinoma. *Cancer Res.*, **48**, 161.

SOUTHERN, E.M. (1975). Detection of specific sequences among DNA fragments separated by gel electrophoresis. *J. Mol. Biol.*, **98**, 503.

SPANDIDOS, D.A., LAMOTHE, A. & FIELD, J.K. (1985). Multiple transcriptional activation of cellular oncogenes in human head and neck solid tumours. *Anticancer Res.*, **5**, 221.

TOWBIN, H., STAEHELIN, T. & GORDON, J. (1979). Electrophoretic transfer of proteins from polyacrylamide gels to nitrocellulose sheets: procedure and some applications. *Proc. Natl Acad. Sci. U.S.A.*, **76**, 4350.

WIGLER, M., SWEET, R., SIM, G.H. and 6 others (1979). Transformation of mammalian cells with genes from procaryotes and eucaryotes. *Cell*, **16**, 777.

XU, Y.H., ISHII, S., CLARK, A.J. and 6 others (1984*a*). Human epidermal growth factor receptor cDNA is homologous to a variety of RNAs overproduced in A431 carcinoma cells. *Nature*, **309**, 806.

XU, Y.H., RICHERT, N., ITO, S., MERLINO, G.T. & PASTAN, I. (1984*b*). Characterization of epidermal growth factor receptor gene expression in malignant and normal human cell lines. *Proc. Natl Acad. Sci. USA*, **81**, 7308.

YAMAMOTO, T., KAMATA, N., KAWANO, H. and 9 others (1986). High incidence of amplification of the epidermal growth factor receptor gene in human squamous carcinoma cell lines. *Cancer Res.*, **46**, 414.

YAMAMOTO, T., NISHIDA, T., MIYAJIMA, N., KAWAI, S., OOI, T. & TOYOSHIMA, K. (1983). The *erb*B gene of avian erythroblastosis virus is a member of the *src* gene family. *Cell*, **35**, 71.

YASUI, W., SUMIYOSHI, H., HATA, J. and 4 others (1988). Expression of epidermal growth factor receptor in human gastric and colonic carcinomas. *Cancer Res.*, **48**, 137.

YOKOTA, J., YAMAMOTO, T., MIYAJIMA, N. and 3 others (1988). Genetic alternations of the c-*erb*B-2 oncogene occur frequently in tubular adenocarcinoma of the stomach and are often accompanied by amplification of the v-*erb*A homologue. *Oncogene*, **2**, 283.